# The Maniac's Wail

**Published:** 1851-10-01

**Authors:** 


### THE MANIAC'S WAIL.

We copy the following beautiful and expressive lines from the columns of the "Examiner." They are from the pen of Mr. Edmund Ollier, and are designated "The Wife-Slayer." We have taken the liberty of altering the title of the poem, as it so accurately and poetically delineates that morbid and insane state of mind which so often irresistibly and blindly impels to acts of suicide and homicide. Our readers will at once recognise in the poem a truthful, beautiful, and affecting sketch. In homicidal insanity the victim is, alas! frequently related to the lunatic by the closest and tenderest ties of consanguinity. A morbid desire to shed human blood, from a conviction that something dreadful *must be done* to relieve the brain of its agonizing pressure, occasionally overpowers all feelings of affection and love. "It *must* be done—it *shall* be done—blood must be shed—my wife—my infant child must perish by my hand before this mental anguish can pass away." Such was the sad description, given to the Editor of this Journal, of the morbid feelings of the most loving and affectionate of husbands and fathers. A gentleman whose mind had been exposed to much distress and anxiety in consequence of pecuniary losses, manifested symptoms which led his family to suspect the approach, if not the actual existence, of mental derangement. One day he was suddenly missing. Search was made in every direction for him, but without avail. Months rolled on, and no tidings of the poor man reached his afflicted wife. After the lapse of nearly half a-year, she was sitting, one beautiful moonlight evening, near the door of her house, when she saw the tall figure of a man, gliding slowly, leisurely, and with measured steps, up an avenue of trees, leading from the mansion to the main road. She watched the somewhat suspicious and singular movements of the stranger, as he occasionally stopped, and, with folded arms, gazed vacantly around him, first at the moon, then at the house, and afterwards at the tall trees skirting each side of the road. As he approached, his wife immediately recognised in the face of the mysterious stranger her long-lost husband, but, alas! he was a lunatic! During the night the wife heard the sound of footsteps stealthily approaching her room. Before she had time to secure the door, it opened, and her husband entered. He made no remark, but after gazing vacantly and sadly round the room, he placed a chair at the head of the bed, and sat quietly down upon it. His wife, in her fright, lost all presence of mind, and made no attempt to speak, or to leave the room, or even the bed. A few minutes had scarcely elapsed, when the lunatic was seen to be gazing rather wildly upon something he held in his hand. He turned his eyes, and fixed them upon the face of his wife, and then they reverted to an object he appeared to have brought with him into the room. To the horror of the poor woman she perceived that her husband had possessed himself of a carving knife. Her first impulse was to seize the instrument and attempt to wrest it from his grasp—her second thought was to escape out of the room; but the great risk which would accompany either movement flashed across her mind, and she resolved to remain quiet and composed, and wait the issue of the anticipated struggle for her life. The lunatic, with an expression denoting the most horrible intentions, gazed for some minutes fixedly and uninterruptedly upon his wife, and then upon the murderous weapon. To her inexpressible joy, she saw a manifest alteration in the character of his countenance. His whole features relaxed, and a sweet smile was seen playing upon his face, and, looking with the affection he was wont to do in happier days gone by, he, with an expression of deep pathos, convulsively exclaimed, "Poor Sally! poor Sally! dear Sally!" Upon this he rose from his seat, and quietly left the room. The wife immediately locked and barricaded the door. In a few minutes the maniac returned, and endeavoured to obtain admission by main force into the apartment. Finding his efforts ineffectual, he returned to his own room. When morning dawned, he was again missing. Under the impression that, failing in his attempts to murder his wife, he might have destroyed himself, a neighbouring pond was dragged, and his dead body discovered.

It was our intention to cite other cases illustrative of that melancholy condition of mind so forcibly described in the poem before us, but want of space forbids us.

"No, no! I did not kill her! No!
I say I will not have it so—
I will not hear it! 'Twas a dream
From which I woke with sudden scream,
And found the sweat upon my brow,
And that dull pain which even now
Is heavy on my heart and brain:—
Oh God! I must have slept again,
And stumble yet through dusky chasms,
Flesh-quakings, and tremendous spasms!

"I have a wife—a dear one.—Nay,
Start not! I have one *still*, I say,—
Or shall, when from this dream I wake.
We were heart-wedded: we did slake
Our miseries in each other's tears,
And grew, through all the strange, sad years,
Quiet in grief's own quietness.
We could walk straight beneath distress,
And make no cry. But want extreme
Seiz'd us; and then—then came this dream!

"Beware! You'd tell me she is dead!
But I will dash my desperate head
Against these walls, before you speak
That cruel word!—Oh foul! You seek
To crush me, seeing I am weak.
You have no touch of human ruth:
You shake me with mere shows of truth
Which *must* be false, or heaven would pass
In shudderings to one formless mass.
Why, look in one another's eyes—
How calm they are! You tell me lies,
Or your own tears would fleck the ground!—
I dreamt it, if this brain is sound.

"I thought I had been out all day,
Wandering, in some half-witted way,

In search of work; and, failing quite,
I came home by the fall of night,
And sat down in my wretched room.
The place was hush'd in ghostly gloom,
And voidness lay upon my eyes,
Until I heard some creature rise
Within the darkness,—and a face
Fell on me like a strange disgrace;—
The face of her whom most I love,
Dead to all thoughts of all above,
Burnt up with drink—a pallid drouth
Around a vague and twitching mouth
That welter'd into speech obscure!
Oh, how could Love itself endure
That loveless sight?—Fierce words upgrew
Between us, raining poisonous dew.
The hot blood sang within my head,
And humm'd through all my veins, and fled
Out of my heart: till, half in fear,
Half rage, I seiz'd a bludgeon near,
And dash'd the face that look'd on mine!
The blood leapt out like awful wine!
My own blood answer'd it. I sought
To beat and crush that face to nought;
And so the human features fell
To crimson blanks—a soul-less shell.
—I felt like one new-born in hell.

"And with a scream from me (not her)
I stagger'd back, and felt a stir
Of gathering crowds, and on my sight
A weight of huge and shoreless night.

"My eyes are fire; but they could weep
Strangely!—I walk even yet in sleep.
Things are not only as they seem:
Men dabble in dark pools of dream,
And shriek themselves awake in bed,
Grey with one night's enormous dread.
Even so shall I. I lean with faith
On what my soul to itself saith.
Yet you who stand about me here
Have almost numb'd me with the fear
That, after all, this thing is real,
And that I kill'd her.—Let me feel
These stony walls and windows barr'd.
Oh, misery! They are firm and hard!

"I wail and wander like a ghost,
Houseless, about a glimmering coast,
Where one lost face makes red the night.
—Oh, lingering dawn! Oh, day! Oh, light!"

